# Parental Neglect and Childhood Obesity Amidst COVID-19: A Developmental Psychopathology Perspective on Health and Developmental Risks

**DOI:** 10.3390/nu16132162

**Published:** 2024-07-07

**Authors:** Silvia Cimino

**Affiliations:** Department of Dynamic, Clinic and Health Psychology, Faculty of Psychology and Medicine, Sapienza University of Rome, 00185 Rome, Italy; silvia.cimino@uniroma1.it

**Keywords:** COVID-19 pandemic, developmental psychopathology, childhood obesity, mental health interventions

## Abstract

The COVID-19 pandemic has profoundly impacted the mental health and developmental trajectories of children and adolescents, catalyzing a range of psychological and behavioral issues due to enforced lockdowns and other restrictions. This text explores these impacts through the lens of developmental psychopathology, which integrates clinical psychology and developmental science to examine the emergence and evolution of psychological disorders across a lifespan. This paper highlights how pandemic-related disruptions have exacerbated conditions such as anxiety and depression and, notably, increased childhood obesity due to changes in lifestyle and reductions in physical activity. The analysis includes a discussion of how isolation has not only restricted access to educational and psychological resources but also increased the risk of parental mental illness and related familial stress, thereby intensifying issues of neglect and their consequent impact on child health. By employing a developmental psychopathology framework, this paper argues for the necessity of targeted interventions that address these complex interplays of genetic, environmental, and psychological factors. Such interventions aim to support children through structured educational and health-oriented strategies, ensuring their well-being amidst the ongoing challenges posed by the pandemic. This approach underscores the importance of early, multifaceted strategies involving parents, educators, and healthcare providers to foster healthier developmental outcomes for children facing unprecedented global health crises.

The COVID-19 pandemic and the measures implemented to limit its spread have had significant unintended consequences, particularly on the mental health and development of children and adolescents. Research highlights that the mental health impacts of the pandemic on children and adolescents are profound and potentially long-lasting. Lockdowns and other restrictions have led to increased reports of anxiety, depression, and other mental health issues among young populations. A systematic review by Panchal et al. [1] noted that anxiety and depression symptoms were notably prevalent, affecting a significant percentage of children during the lockdown periods. These psychological issues were compounded by irritability, anger, and a general increase in mental distress, as posited by Fegert et al. [2], who emphasized that the isolation measures not only limited access to regular educational and psychological support systems but also heightened the risk of parental mental illness, domestic violence, and child maltreatment. This was particularly challenging for children with special needs or pre-existing mental health conditions, who found the pandemic period especially disruptive [2]. The narrative review by Miranda et al. [3] also pointed out that while some supportive strategies emerged during the pandemic, their effectiveness remains uncertain, particularly in addressing long-term mental health outcomes. Children respond to stress differently depending on their developmental stage, and high rates of post-traumatic symptoms were identified, suggesting a critical area for targeted mental health interventions. Throughout the COVID-19 crisis, an intricate relationship between parental neglect and the escalating issue of childhood obesity emerged and was examined through the lens of developmental psychopathology.

Developmental psychopathology [4,5] is an interdisciplinary field that examines the development of psychological disorders within a life-span framework. This field integrates principles from clinical psychology and developmental science to understand how certain maladaptive behaviors or mental health issues can arise and change throughout a person’s life. Originating in the late 20th century, developmental psychopathology emerged from the realization that adult psychopathology could often be traced back to developmental processes that went awry during childhood. Scholars like Norman Garmezy, Michael Rutter, and Dante Cicchetti were pivotal in its foundation, focusing on risk factors, resilience, and the interplay between nature and nurture in the development of psychopathological conditions. This theoretical–clinical framework places significant emphasis on understanding how different trajectories of development can lead to mental health outcomes. It considers the influence of biological, psychological, and socio-environmental factors that interact at various stages of a person’s development. By focusing on these interactions, the field seeks to identify why individuals diverge from typical developmental pathways, which can lead to the onset of disorders. For instance, Rutter [4] highlighted the importance of sensitive periods in development, where certain experiences have profound effects on developmental outcomes, suggesting that timing of risk exposure can significantly affect mental health.

A core principle of this field is that maladaptive and adaptive developments are not distinct pathways but are interwoven across the lifespan. This concept is reflected in the work of Cicchetti and Cohen [5], who articulate that adaptability and vulnerability to mental illness are influenced by earlier developmental processes and subsequent experiences. Their work underscores the importance of a dynamic approach to studying psychopathology, one that accounts for changes across time and differing contexts.

Furthermore, developmental psychopathology focuses on resilience and risk factors. It examines how certain environments, genetic predispositions, and coping mechanisms influence one’s ability to handle stress or overcome adversity. For example, research by Masten et al. [6] into resilience in development provides a framework for studying how children overcome adversities, emphasizing the role of protective factors that help mitigate risk.

In short, developmental psychopathology is a vital field that contributes to our understanding of how psychological disorders can develop and change throughout a person’s life. It integrates findings from various disciplines to offer a comprehensive approach to mental health, emphasizing the necessity of considering a wide range of influences and their interaction over time. This perspective not only helps in diagnosing and treating mental disorders but also offers a blueprint for preventive measures that can be implemented at critical stages of development.

This viewpoint sheds light on how the pandemic’s compounded pressures amplified health and developmental risks. The upheaval caused by the pandemic has reshaped children’s social and educational environments, emphasizing the influence of varied forms of parental neglect—emotional, educational, or physical—on the health outcomes of children, with a notable impact on the prevalence of overweight and obesity issues. Studies indicate a rise in children’s screen time, diminished physical activity, and irregular sleep patterns during the pandemic, highlighting the role of changed environmental conditions on children’s health behaviors [7]. Furthermore, the pandemic’s influence on nutritional habits and the increased likelihood of excessive weight gain in children underscore the drastic alterations in daily routines that favor a sedentary lifestyle and unhealthy eating habits [8]. In fact, many studies observed an increase in the consumption of home-cooked meals, snacks, sweets, and bakery products, while the intake of fast food and soft drinks decreased. These dietary changes were mixed in their health implications; while home-cooked meals might be healthier, the increase in snacks and sweets can lead to adverse health outcomes. For example, the increased intake of snacks and sweets is associated with higher risks of obesity and related metabolic disorders, which in turn can exacerbate mental health issues such as anxiety and depression [9]. Additionally, emotional eating driven by stress and anxiety during confinement was linked to the increased consumption of sugary and fatty foods, contributing to weight gain and poor mental health outcomes [10]. The altered eating patterns, combined with reduced physical activity and increased screen time, further amplified these negative health effects, underscoring the need for targeted interventions to promote healthy eating and physical activity during such periods [11]. School closures are pinpointed as a significant factor for weight gain in children, removing the structured support for regular exercise and nutritious eating habits [12]. These elements collectively present a complex challenge, where neglect in various forms, intensified by pandemic conditions, significantly affects the increasing trends in childhood overweight and obesity.

In the wake of COVID-19, public health, mental health, and child development face intersecting challenges, notably the surge in rates of overweight and obesity among children during the pandemic. Viewing this issue through a developmental psychopathology framework emphasizes neglect’s pivotal role in intensifying these challenges, affecting children’s physical, emotional, and developmental well-being. The lockdown period has notably led to significant increases in body weight and BMI among school-aged children and adolescents, underscoring the negative effects of the pandemic on children’s physical health [13]. Additionally, as said above, the closure of schools during the pandemic was directly linked to a heightened risk of weight gain in children, reflecting the direct impact of disrupted regular school activities on obesity rates [14]. This complex scenario underscores the need for comprehensive strategies to address the multi-dimensional aspect of neglect and its influence on children’s health amidst the ongoing pandemic.

The pandemic has heightened parental stress levels, possibly leading to both physical and emotional neglect. Physical neglect, manifested as poor nutrition and inadequate structured physical activity due to lockdown measures, directly heightens the risk of children becoming overweight. Emotional neglect, seen through a lack of emotional support and engagement, may intensify stress-related eating and inactive behaviors in children, contributing to weight gain. The COVID-19 pandemic’s disruption to daily life has significantly affected health behaviors, stress levels, and the financial and food security of families with young children, indicating the wider scope of physical and emotional neglect’s potential effects on child nutrition and activity patterns [15,16]. Parental stress and perceived risk during the pandemic negatively correlated with children’s physical activity and well-being, highlighting emotional neglect’s indirect effects on children’s lifestyle choices and mental health [17]. These observations underscore the linkage between parental stress, neglect, and the negative outcomes on children’s physical and emotional well-being during the COVID-19 pandemic.

The transition to remote learning and the subsequent lack of physical education and extracurricular activities embody educational neglect, directly affecting children’s physical health. The diminished opportunities for structured physical activity contribute to a sedentary lifestyle, raising the risk of becoming overweight. The global shift to remote learning has significantly affected children’s physical activity and mental health outcomes, with school-aged children experiencing notable reductions in physical activity since the transition, highlighting the challenge of providing adequate physical activity opportunities during this period [18,19,20]. Moreover, studies during the pandemic confirmed the diminished quality and duration of physical education classes in remote schooling, revealing a significant decline in children’s physical/sports activity and motor functions, which underscores the inadequate conditions for children’s healthy development during remote schooling [21,22]. These findings emphasize the need to bridge the gap in physical activity and health education for children during remote learning periods to mitigate sedentary lifestyle-associated risks.

The developmental psychopathology perspective brings to light the significance of intergenerational transmission within the context of neglect and obesity [23]. Parents who have endured neglect or obesity in their youth might unintentionally pass on behaviors that elevate their children’s risk of becoming overweight, particularly amid the pandemic’s heightened stress and disruptions. Research on intergenerational stress illustrates that stress during pregnancy can result in social deficits and depressive-like behaviors in offspring, suggesting a dual-hit stress mechanism involving both prenatal and early-life parenting environments. These insights affirm the intricate relationship between genetic, environmental, and behavioral factors in the intergenerational transmission of stress and behaviors related to obesity [24,25]. Furthermore, the link between maternal lifetime stressors and the depressive symptoms of high-risk adolescent daughters through the daughters’ lifetime stressors underscores stress transmission and its implications for subsequent generations, especially in terms of neglect and the heightened risk of obesity [26,27]. These observations highlight the necessity for interventions that tackle the foundational causes of stress and obesity, taking into account the intergenerational patterns that may intensify these issues.

In addressing these issues, a developmental psychopathology lens underscores the criticality of early, targeted interventions to disrupt the cycle of neglect and obesity. Potential strategies include providing support to parents and caregivers, enhancing access to resources, delivering mental health interventions, and initiating educational initiatives. These evidence-based approaches underscore the multifaceted strategy required to navigate the interconnected issues of neglect and childhood obesity, emphasizing the importance of early, comprehensive interventions involving parents, caregivers, educators, and healthcare providers in the pursuit of healthier developmental trajectories for children amidst unprecedented challenges [28,29,30].

Addressing the complex interplay between neglect and overweight in children, especially during the COVID-19 pandemic, necessitates a comprehensive approach that is responsive to the multifaceted nature of these challenges. The pandemic has not only disrupted the lives of children and families but also presented an opportunity to reevaluate and reinforce the systems of support that can prevent and mitigate the effects of neglect and contribute to healthier outcomes for children.

Parental and caregiver support is paramount in addressing physical and emotional neglect. By providing resources and education on stress management, nutrition, and the importance of physical activity, we can empower parents and caregivers to foster environments that support healthy development. This involves not only direct educational efforts but also support systems that reduce the stressors contributing to neglect, such as financial assistance programs, access to mental health services, and community support networks [31].

Improving access to nutritious food and physical activity opportunities is critical, particularly in communities where these resources are scarce. This can involve initiatives like community gardens, subsidized healthy meal programs for children, and safe, accessible spaces for physical activity. During the pandemic, when traditional avenues for physical education and activity were disrupted, creative solutions such as virtual exercise classes or socially distanced outdoor activities helped fill the gap.

The psychological well-being of both children and their parents or caregivers plays a critical role in addressing neglect and its consequences. Offering mental health services, including psychological support and therapy, can provide families with the tools to manage stress, address emotional neglect, and foster healthier relationships. These services can be delivered through telehealth platforms to increase accessibility, especially during times when in-person services may be limited [32,33,34].

Schools are also crucial in supporting children’s physical and mental health. Beyond providing education, they can offer structured physical activities, nutrition programs, and mental health resources. Ensuring that remote or hybrid learning models include components that address physical education and social–emotional learning can help mitigate the impacts of educational neglect.

At a broader level, policy changes and community actions can support the systemic changes needed to address neglect and its impacts on child health. This includes advocating for policies that support family well-being, such as paid parental leave, affordable healthcare, and accessible mental health services. Community efforts to raise awareness and create supportive networks can also play a vital role in preventing neglect and promoting healthy development [35,36,37].

The COVID-19 pandemic has highlighted and intensified the challenges of parental neglect and its impact on childhood overweight and obesity. By adopting a comprehensive approach that addresses the needs of children and families from a developmental psychopathology perspective, we can work towards mitigating these issues. This requires the collaboration of healthcare providers, educators, policymakers, and communities to create a supportive environment that promotes the well-being and healthy development of all children [38,39]. Through targeted interventions, support for parents and caregivers, and systemic changes, we can strive to ensure that children have the opportunity to thrive, even in the face of unprecedented challenges.
